# Hematoma enlargement characteristics in deep versus lobar intracerebral hemorrhage

**DOI:** 10.1002/acn3.51001

**Published:** 2020-03-04

**Authors:** Jochen A. Sembill, Joji B. Kuramatsu, Stefan T. Gerner, Maximilian I. Sprügel, Sebastian S. Roeder, Dominik Madžar, Manuel Hagen, Philip Hoelter, Hannes Lücking, Arnd Dörfler, Stefan Schwab, Hagen B. Huttner

**Affiliations:** ^1^ Department of Neurology University of Erlangen‐Nürnberg Schwabachanlage 6 Erlangen 91054 Germany; ^2^ Department of Neuroradiology University of Erlangen‐Nürnberg Schwabachanlage 6 Erlangen 91054 Germany

## Abstract

**Objective:**

Hematoma enlargement (HE) is associated with clinical outcomes after supratentorial intracerebral hemorrhage (ICH). This study evaluates whether HE characteristics and association with functional outcome differ in deep versus lobar ICH.

**Methods:**

Pooled analysis of individual patient data between January 2006 and December 2015 from a German‐wide cohort study (RETRACE, I + II) investigating ICH related to oral anticoagulants (OAC) at 22 participating centers, and from one single‐center registry (UKER‐ICH) investigating non‐OAC‐ICH patients. Altogether, 1954 supratentorial ICH patients were eligible for outcome analyses, which were separately conducted or controlled for OAC, that is, vitamin‐K‐antagonists (VKA, *n* = 1186) and non‐vitamin‐K‐antagonist‐oral‐anticoagulants (NOAC, *n* = 107). Confounding was addressed using propensity score matching, cox regression modeling and multivariate modeling. Main outcomes were occurrence, extent, and timing of HE (>33%/>6 mL) and its association with 3‐month functional outcome.

**Results:**

Occurrence of HE was not different after deep versus lobar ICH in patients with non‐OAC‐ICH (39/356 [11.0%] vs. 36/305 [11.8%], *P* = 0.73), VKA‐ICH (249/681 [36.6%] vs. 183/505 [36.2%], *P* = 0.91), and NOAC‐ICH (21/69 [30.4%] vs. 12/38 [31.6%], *P* = 0.90). HE extent did not differ after non‐OAC‐ICH (deep:+59% [40–122] vs. lobar:+74% [37–124], *P* = 0.65), but both patients with VKA‐ICH and NOAC‐ICH showed greater HE extent after deep ICH [VKA‐ICH, deep: +94% [54–199] vs. lobar: +56% [35–116], *P* < 0.001; NOAC‐ICH, deep: +74% [56–123] vs. lobar: +40% [21–49], *P* = 0.001). Deep compared to lobar ICH patients had higher HE hazard during first 13.5 h after onset (Hazard ratio [HR]: 1.85 [1.03–3.31], *P* = 0.04), followed by lower hazard (13.5–26.5 h, HR: 0.46 [0.23–0.89], *P* = 0.02), and equal hazard thereafter (HR: 0.96 [0.56–1.65], *P* = 0.89). Odds ratio for unfavorable outcome was higher after HE in deep (4.31 [2.71–6.86], *P* < 0.001) versus lobar ICH (2.82 [1.71–4.66], *P* < 0.001), and only significant after small‐medium (1st volume‐quarter, deep: 3.09 [1.52–6.29], *P* < 0.01; lobar: 3.86 [1.35–11.04], *P* = 0.01) as opposed to large‐sized ICH (4th volume‐quarter, deep: 1.09 [0.13–9.20], *P* = 0.94; lobar: 2.24 [0.72–7.04], *P* = 0.17).

**Interpretation:**

HE occurrence does not differ among deep and lobar ICH. However, compared to lobar ICH, HE after deep ICH is of greater extent in OAC‐ICH, occurs earlier and may be of greater clinical relevance. Overall, clinical significance is more apparent after small–medium compared to large‐sized bleedings.

## Introduction

Hematoma enlargement (HE) represents a major predictor of clinical outcome after supratentorial intracerebral hemorrhage (ICH) and is associated with antithrombotic treatment, blood pressure levels, and cranial imaging time window‐related baseline hematoma volumes.^1^, ^2^, ^3^, ^4^, ^5^, ^6^ While randomized trials targeting hemostasis and blood pressure management have demonstrated reduced occurrence and extent of HE, these interventions did not translate to improved clinical outcomes for the overall ICH patients.^7^, ^8^, ^9^, ^10^, ^11^

However, the occurrence, extent, and timing of HE may vary according to ICH location, that is, deep versus lobar ICH, given a different underlying etiology and pathophysiology, and thus influence outcome differently.^12^, ^13^, ^14^ In this pooled individual participant data analysis, conducted separately for patients with and without oral anticoagulation associated (OAC‐) ICH, we tested the hypotheses that (1) HE occurs more frequently, earlier, and at a larger extent in deep versus lobar ICH and (2) HE is of greater clinical significance in deep versus lobar ICH location. Hypotheses are pathophysiologically based on the assumption that deep ICH represent hypertension‐induced ruptures of penetrating arteries in immediate proximity of passing pyramidal tracts crucial for patient’s motor function, whereas lobar ICH are frequently caused by cerebral amyloid angiopathy (CAA), potentially leading to rupture under lower pressure resulting in less hematoma enlargement and more likely cortical deficits underrepresented in common outcome assessments.^15^, ^16^

## Methods

### Study design

This observational cohort study pooled individual patient data from both parts of the retrospective German‐wide RETRACE‐program (“geRman‐widE mulTicenter Analysis of oRal Anticoagulation associated intraCerebral hEmorrhage”) which recruited patients with OAC‐ICH at 22 tertiary care centers across Germany from January 01, 2006 until December 31, 2010(part‐1,NCT01829581)^3^ and from 01 January 2011 until 31 December 2015 (part‐2, NCT03093233),^17^ as well as from the prospective single‐center UKER‐ICH registry recruiting spontaneous ICH patients admitted to the University Hospital Erlangen (01 January 2006 until 31 December 2015 [NCT03183167]), as previously described.^18^, ^19^, ^20^ Conduction of the study was approved by local ethics committees and institutional review boards based on the central vote from Friedrich‐Alexander‐University Erlangen‐Nuremberg, Germany (Re.No‐4409&30_16B,115_17B).^18^ Unless waived by local ethics committees individual consent was obtained by all patients or legal representatives.^18^

### Data acquisition

#### Demographic, clinical, and laboratory parameters

Data on demographics, prior medical history and medication, clinical admission status, in‐hospital parameters and measurements, and laboratory data, were obtained as previously described.^3^ We differentiated primary spontaneous ICH in the absence of therapeutic anticoagulation (non‐OAC‐ICH) from ICH related to oral anticoagulants. OAC‐ICH was defined as either ICH on effective treatment with vitamin‐K‐antagonists (VKA) (INR > 1.5 on hospital admission) or ICH on known treatment with non‐vitamin‐K‐antagonist oral anticoagulant (NOAC) at symptom onset.^3^, ^17^, ^21^, ^22^ We defined early care limitations as care limitation employed during first 24 h after hospital admission.^23^

#### Imaging

Imaging data were analyzed by neuroradiologist blinded to clinical data.^20^ Diagnosis of spontaneous ICH and related imaging parameters, that is, hematoma volume and location, intraventricular hemorrhage (IVH), and time from symptom onset until imaging, were assessed on first cranial imaging after symptom onset as well as on follow‐up imaging. Secondary ICH caused by etiologies such as aneurysms, arteriovenous malformations, tumor, trauma, or coagulopathies other than anticoagulation were excluded.^3^ Hematoma volume was calculated by validated ABC methods.^24^, ^25^, ^26^ We defined ICH involving the thalamus, basal ganglia, internal capsule, or deep periventricular white matter as deep ICH, while ICH originating at the cortex and cortical‐subcortical junction was defined as lobar ICH.^14^ In case of large ICH affecting both regions, location was scored according to the location that hemorrhage most likely originated from [23, 27].

### Outcomes

#### Primary outcome

The primary outcome was the occurrence of HE defined according to the most commonly used approach as an increase in ICH volume of more than 33% or 6 mL from initial to follow‐up imaging.^5^, ^9^

#### Secondary outcomes

Secondary outcomes included the extent of hematoma volume change in case of HE, the time from symptom onset until radiological HE detection, and mortality and functional outcome after 3 months in relation to HE occurrence. We assessed the functional outcome using the modified Rankin Scale (mRS, range 0–6, higher scores indicate worse outcome; 6 indicates death), dichotomized as favorable (0–3) and unfavorable (4–6) outcome.^3^

### Statistics

Statistical analyses were performed using the SPSS 21.0 software package (http://www.spss.com) or R2.12.0 (http://www.r-project.org).^28^ We conducted two‐sided statistical tests, setting the significance level at *α* = 0.05.^28^ Data distribution was evaluated using the Kolmogorov–Smirnov test.^29^ We present normally distributed data as mean (±standard deviation), analyzed using the Student's t‐test, and non‐normally distributed data as median (interquartile range), compared using the Mann–Whitney *U* test.^28^ We performed the Pearson’s chi‐squared or Fisher´s exact tests to analyze frequency distribution of categorized variables.^29^ To minimize accumulation of type‐1 errors, univariate analyses were corrected for multiple comparisons using the Holm’s sequential Bonferroni procedure.^19^

For comparison of HE occurrence in deep versus lobar ICH location (primary outcome), we separately analyzed patients with non‐OAC‐ICH, VKA‐ICH, and NOAC‐ICH to minimize confounding by established larger ICH volumes, higher HE rates, and need for reversal treatment in patients with prior anticoagulation intake.^17^, ^30^ To further address potential confounding by varying baseline characteristics and time points of imaging, we additionally performed a parallel, balanced, nearest‐neighbor (1:1, caliper 0.1) propensity score matching (PSM). Due to limited numbers of patients taking NOAC, we combined cohorts of NOAC‐ICH and VKA‐ICH for PSM‐analyses. PSM was performed to adjust for imbalances showing a statistical trend (*P* < 0.1) in known HE predictors, that is, ICH volume, antiplatelet therapy, initial systolic blood pressure, time from symptom onset until 1st and until 2nd imaging, and reversal treatment with prothrombin complex concentrate (PCC) in OAC‐ICH‐patients, as well as in parameter with major clinical relevance, that is, age and Glasgow Coma Scale (GCS) in non‐OAC‐ICH patients. Association of patients excluded from PSM with hematoma enlargement according to ICH location was tested for interaction. Presence of IVH was not included into PSM due to its inherent association with deep ICH^12^, ^14^ caused by immediate proximity of the ventricular system, resulting in location‐specific interaction of hematoma size with IVH. We therefore corrected analyses by additional adjustment in multivariable modeling, as well as cox regression modeling as described below.

The extent of HE (secondary outcome) was compared in deep versus lobar ICH patients experiencing HE as defined above, conducting separate analyses on patients with non‐OAC‐ICH, VKA‐ICH, and NOAC‐ICH as well as in cohorts after PSM, that is, matched patients with non‐OAC‐ICH and OAC‐ICH.

Timing from symptom onset until radiological HE detection (secondary outcome) in deep compared to lobar ICH was analyzed using PSM cohorts, combining Non‐OAC‐ICH and OAC‐ICH patients. We graphically compared time points of HE detection since symptom onset by Kaplan–Meier estimator. Using multivariable COX regression modeling with additional adjusting for IVH and OAC, we calculated adjusted hazard ratio (HR) estimates for each hour after symptom onset derived from patient clusters (HR estimate at the median of a 5‐h interval, i.e., HR estimate at hour 10 calculated using the interval from hours 8 to 12).^19^ To correct for overestimation, HR estimates were weighted and smoothed by the method of moving averages.^19^ The intercept of the median with the HR of 1 allowed identification of a time interval at which patients were at increased risk to experience HE in relation to ICH location.^19^ Crude HE rates over time were compared using the chi‐squared test.

The association of HE with mortality and functional outcome (secondary outcome) was assessed by comparison of mortality and dichotomized mRS in patients with and without HE in both deep and lobar ICH cohorts using complete case analysis including 1703 patients (87.2%). To adjust for baseline confounding, we performed multivariable regression analyses using combined cohorts of both non‐OAC‐ICH and OAC‐ICH patients and adjusted analyses for imbalanced outcome predictors, that is, age, OAC, GCS, initial ICH volume, and IVH. To investigate the association of HE with mortality and functional outcome in relation to the initial ICH volume, we computed regression analyses corrected as mentioned above after splitting cohorts according to initial ICH volume quartiles. Adjusted odds ratios (OR) were graphically compared using forest plots.

## Results

### Study population and baseline characteristics

**Figure 1:**
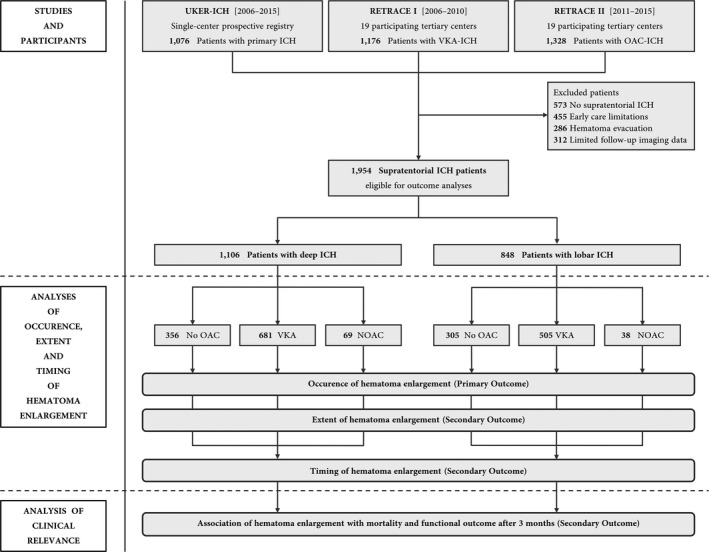
Study flowchart. Altogether, individual level data from 3,580 spontaneous ICH patients were analyzed to identify 1,954 supratentorial ICH patients eligible for outcome analyses. Data were provided by two parts of a German‐wide observational studies (RETRACE I and II) conducted at 22 participating tertiary centers, and by one single‐center university hospital registry.

For the present study, a total of 3,580 pooled spontaneous ICH patients were available of whom patients with infratentorial ICH (*n* = 573), early care limitations (*n* = 455), surgical hematoma evacuation (*n* = 286), and those with limited data on imaging parameters or no follow‐up imaging (*n* = 312) were excluded (Fig. 1). Of 1,954 eligible supratentorial ICH patients (661 with non‐OAC‐ICH, 1,186 with VKA‐ICH, and 107 with NOAC‐ICH), 1,106 had deep ICH and 848 patients lobar ICH, for baseline characteristics (see Table S1).

Among patients with non‐OAC‐ICH (Table 1A), those with deep ICH compared to lobar ICH were younger (68 y [58–77] vs. 74 y [68–81], *P* < 0.001), had smaller ICH volumes (8.8 mL [3.4–20.2] vs. 17.9 mL [6.3–34.9], *P* < 0.001), more frequent IVH (219/356 [61.5%] vs. 83/305 [27.2%], *P* < 0.001), lower GCS levels (13 [8–15] vs. 14 [12–15], *P* < 0.001) were more often on antiplatelet treatment (12/356 [31.6%] vs. 33/305 [10.8%], *P* < 0.001), and received initial and control imaging earlier after onset (243 min [106–512] vs. 445 min [208–1318], *P* < 0.001; 28 h [21–43] vs. 35 h [22–61], *P* < 0.001).

**Table 1:** Clinical characteristics of patients with deep versus lobar ICH.

Characteristics	(A) Non‐OAC‐ICH (*n* = 661)			(B) VKA‐ICH (*n* = 1186)			(C) NOAC‐ICH (*n* = 107)		
	Deep (*n* = 356)	Lobar (*n* = 305)	*P* value	Deep (*n* = 681)	Lobar (*n* = 505)	*P* value	Deep (*n* = 69)	*P* Lobar (*n* = 38)	*P* value
Age^†^ (yrs)	68 (58–77)	74 (68–81)	<0.001	76 (70–81)	76 (70–81)	0.84	78 (73–82)	78 (73–84)	0.56
Sex* (♀)	149 (41.9%)	141 (46.2%)	0.26	244 (35.8%)	210 (41.6%)	0.04^5^	28 (40.6%)	21 (55.3%)	0.14
Pre‐mRS^†1^	0 (0–2)	1 (0–2)	0.009	0 (0–1)	0 (0–2)	0.824	1 (0–2)	1 (0–2)	0.71
Prior medical history									
Hypertension*	321 (90.4%)	246 (80.7%)	<0.001	599 (88.0%)	440 (87.1%)	0.67	64 (92.6%)	34 (89.5%)	0.72
Diabetes mellitus*	97 (27.3%)	76 (24.9%)	0.50	222 (32.6%)	160 (31.7%)	0.74	22 (31.9%)	10 (26.3%)	0.55
Prior ischemic stroke or TIA*	67 (18.9%)	50 (16.4%)	0.42	200 (29.4%)	135 (26.7%)	0.32	25 (36.2%)	7 (18.4%)	0.05
Prior ICH or major bleeding*	23 (6.5%)	54 (17.7%)	<0.001	50 (7.3%)	44 (8.7%)	0.39	9 (13.0%)	4 (10.5%)	0.77
Congestive heart failure*	46 (13.2%)	30 (9.8%)	0.22	125 (18.4%)	98 (19.4%)	0.65	15 (21.7%)	9 (23.7%)	0.82
Renal insufficiency*	55 (15.4%)	25 (8.2%)	0.004	173 (25.4%)	140 (27.7%)	0.37	13 (18.8%)	7 (18.4%)	1.000
Antiplatelet use*	112 (31.6%)	33 (10.8%)	<0.001	57 (8.4%)	58 (11.5%)	0.07	10 (14.5%)	7 (18.4%)	0.60
On admission status									
Glasgow coma scale^†2^	13 (8–15)	14 (12–15)	<0.001	14 (11–15)	14 (12–15)	0.13	14 (9–15)	14 (11–15)	0.73
ICH score^†3^	1 (1–2)	1 (0–2)	0.001	1 (0–2)	1 (0–2)	0.90	1 (1–2)	1 (1–2)	0.46
Systolic blood pressure^†^ (mmHg)	170 (159–190)	160 (146–186)	0.05^5^	169 (150–190)	160 (143–180)	<0.001	174 (150–188)	159 (138–183)	0.06
Imaging									
Symptom onset – first imaging^†^ (min)	243 (106–512)	445 (208–1318)	<0.001	115 (78–257)	210 (99–441)	<0.001	178 (79–407)	213 (105–467)	0.24
Symptom onset – second imaging^†^ (h)	28 (21–43)	35 (22–61)	<0.001	20 (9–31)	25 (13–44)	<0.001	26 (13–39)	23 (9–39)	0.50
ICH volume^†^ (mL)	8.8 (3.4–20.2)	17.9 (6.3–34.9)	<0.001	9.7 (4.0–18.3)	20.9 (7.3–40.3)	<0.001	9.2 (3.6–16.3)	22.2 (8.4–40.1)	0.002
Intraventricular hemorrhage*	219 (61.5%)	83 (27.2%)	<0.001	287 (42.1%)	147 (29.1%)	<0.001	34 (49.3%)	10 (26.3%)	0.02^5^
Reversal treatment									
Any reversal treatment*	N/A	N/A	N/A	671 (98.5%)	485 (96.0%)	0.007^5^	54 (78.3%)	28 (73.7%)	0.59
Admission – reversal^†^ (min)				95 (60–166)	109 (66–209)	0.004^5^	96 (68–141)	86 (44–165)	0.53
PCC*				604 (88.7%)	417 (82.6%)	0.003^5^	52 (75.4%)	28 (73.7%)	0.94
FFP*				32 (4.7%)	40 (7.9%)	0.02^5^	0 (0.0%)	1 (2.6%)	0.36
Konakion*				597 (87.7%)	428 (84.8%)	0.15	4 (5.8%)	1 (2.6%)	0.65
Coagulation parameters									
INR on admission	1.02 (0.97–1.10)	1.03 (0.98–1.09)	0.78	2.60 (2.18–3.18)	2.69 (2.18–3.47)	0.04^5^	1.27 (1.11–1.58)	1.30 (1.14–1.76)	0.30
1st INR after reversal^4^	N/A	N/A	N/A	1.27 (1.15–1.43)	1.32 (1.18–1.57)	<0.001	1.17 (1.04–1.24)	1.17 (1.06–1.25)	0.80
Length of stay (d)	14 (8–21)	10 (7–14)	<0.001	12 (7–18)	10 (7–17)	0.005^5^	12 (8–17)	13 (9–18)	0.54

d, days; FFP, Fresh frozen plasma; h, hours; ICH, intracerebral hemorrhage; INR, international normalized ratio; m, minutes; N/A, not applicable; NOAC, Non‐vitamin K antagonist oral anticoagulants; PCC, prothrombin complex concentrate; TIA, transient ischemic attack; yrs, years.

^1^ Modified Rankin Scale, range 0–6, from no disability to death.

^2^ Glasgow coma scale, range 3–15, from coma to alertness.

^3^ ICH score, range 0–6, from low to high short‐term mortality risk.

^4^ First value of in‐hospital monitoring after reversal treatment, if appropriate.

^5^ Not significant after performance of Holm’s sequential Bonferroni correction.

* *n* (%).

^†^ Median (Interquartile range, 25th–75th percentile).

Among patients with VKA‐ICH (Table 1B), those with deep ICH compared to lobar ICH had smaller ICH volumes (9.7 mL [4.0–18.3] vs. 20.9 mL [7.3–40.3], *P* < 0.001), more frequent IVH (287/681 [42.1%] vs. 147/505 [29.1%], *P* < 0.001), received initial and control imaging earlier after onset (115 min [78–257] vs. 210 min [99–441], *P* < 0.001; 20 h [9–31] versus 25 h [13–44], *P* < 0.001), had higher systolic blood pressure levels (169 mmHg [150–190] vs. 160 mm Hg [143–180], *P* < 0.001), and lower INR levels after reversal (1.27 [1.15–1.43] vs. 1.32 [1.18–1.57], *P* < 0.001).

Among patients with NOAC‐ICH (Table 1C), those with deep ICH compared to lobar ICH differed significantly by smaller ICH volumes (9.2 mL [3.6–16.3] vs. 22.2 mL [8.4–40.1], *P* < 0.01).

### Primary outcome

**Figure 2:**
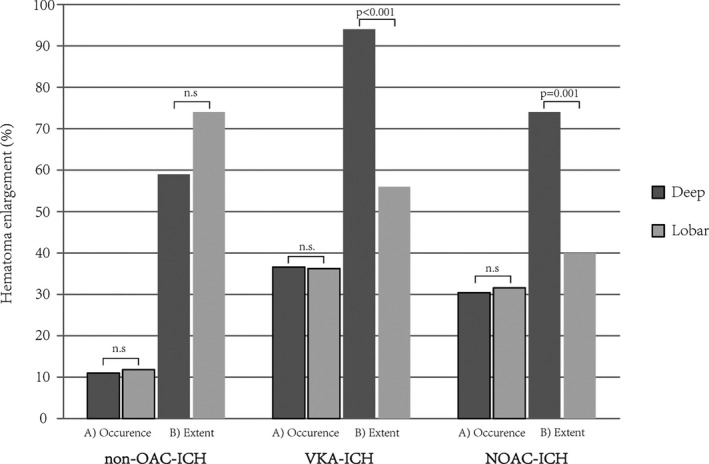
Occurrence and extent of intracerebral hematoma enlargement. Occurrence (A) and extent (B) of hematoma enlargement in patients with deep compared to lobar ICH. Hematoma enlargement was defined as an increase in ICH volume of more than 33% or 6 mL from initial to follow‐up imaging. The extent of hematoma enlargement, that is, percentage ICH volume increase, was compared in patients actually suffering hematoma enlargement. Separate analyses were conducted for patients with non‐OAC‐ICH (*n* = 661), VKA‐ICH (*n* = 1186), and NOAC‐ICH (*n* = 107). Abbreviations: ICH, Intracerebral hemorrhage; NOAC, non‐vitamin‐K‐antagonist oral anticoagulant; n.s., not significant; OAC, Oral anticoagulation; VKA, vitamin‐K‐antagonist.

The occurrence of HE did not differ between deep and lobar location in patients with non‐OAC‐ICH (39/356 [11.0%] vs. 36/305 [11.8%], *P* = 0.73), VKA‐ICH (249/681 [36.6%] vs. 183/505 [36.2%], *P* = 0.91) and NOAC‐ICH (21/69 [30.4%] vs. 12/38 [31.6%], *P* = 0.90), Figure 2A. To verify that results were not confounded by baseline imbalances, we repeated analyses after PSM, for exploratory investigation of parameters associated with hematoma enlargement and evenly balanced cohorts post‐matching see Tables S1 and S3. Standardized mean differences and propensity scores before and after matching procedure are shown in Figure S1. Sensitivity analyses of patients excluded from PSM documented no significant interaction between ICH location and HE (Tables S4 and S5). PSM‐based analyses confirmed no varying HE rates among patients with deep or lobar non‐OAC‐ICH (17/135 [12.6%] vs. 16/135 [11.9%], *P* = 0.86) and OAC‐ICH (55/157 [35.0% vs. 62/157 [39.5%], *P* = 0.41). Further adjustment for IVH using multivariate modeling led to no differently increased odds for HE according to ICH location (OR [95% confidence interval], non‐OAC‐ICH: 0.87 [0.39–1.92], *P* = 0.87; OAC‐ICH: 0.90 [0.56–1.45], *P* = 0.67).

### Secondary outcomes

#### Extent of HE in deep versus lobar ICH

To compare the extent of HE in deep versus lobar position, we investigated all patients who actually suffered HE (Fig. 2B). We observed no significant difference among the extent of HE according to ICH location in patients with non‐OAC‐ICH (deep: +59% [40–122] vs. lobar: +74% [37–124], *P* = 0.65). Both patients with VKA‐ICH and NOAC‐ICH showed greater extent of HE after deep compared to lobar ICH (VKA‐ICH, deep: +94% [54–199] vs. lobar: +56% [35–116], *P* < 0.001; and NOAC‐ICH, deep: +74% [56–123] vs. lobar: +40% [21–49], *P* = 0.001). Results were confirmed in analyses on PSM cohorts (Non‐OAC‐ICH, deep: +51% [42–209] vs. lobar: +104% [48–250], *P* = 0.33 and OAC‐ICH, deep: +99% [60–230] vs. lobar: +60% [40–146], *P* = 0.04).

#### Time‐dependent occurrence of HE in deep versus lobar ICH

**Figure 3:**
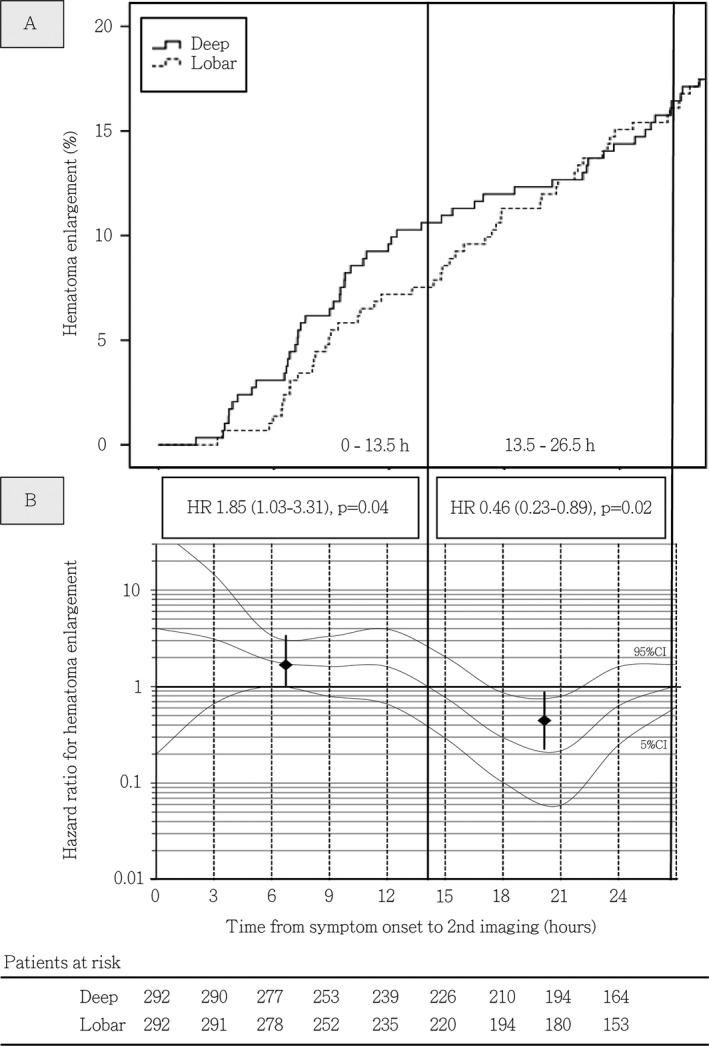
Incidence rates and time‐dependent hazard ratios for hematoma enlargement in patients with deep versus lobar ICH. (A) Incidence rates of hematoma enlargement detected through control imaging during the hyperacute course of deep and lobar ICH management in propensity‐matched cohorts. (B) Time‐dependent hazard ratios for hematoma enlargement in deep versus lobar ICH patients. Adjusted COX proportional hazard models were calculated for propensity‐matched cohorts with additional adjustment for intraventricular hemorrhage and prior oral anticoagulation to visualize the association between time since symptom onset and detection of hematoma enlargement by control imaging in patients dichotomized according to supratentorial ICH location. Hazard ratio estimates (y‐axis) for deep ICH patients were calculated at each hour since symptom onset using time‐patient‐clusters (HR estimate at the median of a 5‐hour interval) of patients with control imaging at a median of the presented hour (x‐axis) and compared with lobar ICH patients with data points within these clusters. To correct for overestimation, we weighted and smoothed hazard ratios by the method of moving averages. The dashed lines indicate time intervals with increased risk for detection of hematoma enlargement identified by the intercept of the adjusted HR median with the HR of 1. Medians and 95% CI displayed as square with whiskers represent hazard ratios for HE in deep compared to lobar ICH during mentioned identified time intervals, that is, 0–13.5 and 13.5–26.5 h. Patients at risk included in both analyses (A + B) are displayed using 3‐hour intervals, showing comparable numbers of patients receiving control imaging at each time point from individual onset of deep or lobar ICH. Abbreviations: CI, Confidence interval; HR, Hazard Ratio; h, hours; ICH, Intracerebral hemorrhage.

We analyzed the timing of radiological HE detection in deep versus lobar ICH patients using combined PSM cohorts to ensure similar propensities for HE occurrence while harmonizing strongly varying time from symptom onset until initial and control imaging to compare HE occurrence over time. Analyzing the first 72 h using multivariable Cox regression modeling with additional adjustment for IVH and OAC, the hazard for HE detection did not differ in deep compared to lobar ICH (HR: 0.95 [0.68–1.33], *P* = 0.76). Focusing on the hyperacute phase of ICH management only, we detected a significantly increased hazard for HE in deep compared to lobar ICH during the first 13.5 h (HR: 1.85 [1.03–3.31], *P* = 0.04), followed by a time period with significantly decreased hazard compared to lobar ICH (13.5–26.5 h, HR: 0.46 [0.23–0.89], *P* = 0.02), and equal hazards thereafter (>26.5 h, HR: 0.96 [0.56–1.65], *P* = 0.89), see Figure 3A for corresponding Kaplan–Meier estimator and Figure 3B for Cox regression modeling. Results were comparable calculating crude HE rates over time (0–13.5 h, deep: 30/58 [51.7%] vs. lobar: 22/65 [33.8%], *P* < 0.05; 13.5–26.5 h, deep: 16/96 [16.7%] vs. lobar: 24/89 [27.0%], *P* = 0.09). To analyze location‐specific confounding of imaging time points by potential emergency imaging related to HE, we performed multivariable modeling showing that time from initial to control imaging was not differently associated with hematoma enlargement among deep and lobar ICH patients (Table S6). Rates of early control imaging, that is, within 12 h after initial imaging, did also not differ (deep: 71/292 [24.3%] vs. lobar: 78/292 [26.7%], *P* = 0.51).

#### Clinical relevance of HE in deep versus lobar ICH

**Figure 4:**
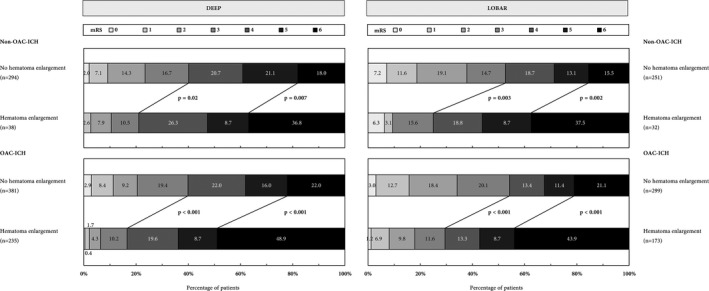
Functional outcome of patients with supratentorial Non‐OAC‐ICH and OAC‐ICH comparing patients with and without hematoma enlargement. Distribution of functional outcome and mortality at 3 months using the modified Rankin Scale (mRS, range 0–6, from 0 = no symptoms, to 5 = severe disability, and 6 = dead). Dichotomized comparison of patients with and without hematoma enlargement. The thick lines separate proportion of patients with favorable (mRS 0–3) and unfavorable (mRS 4–6) outcome as well as patients with and without 3‐month mortality. Abbreviations: ICH, Intracerebral hemorrhage; mRS, modified Rankin Scale; OAC, Oral anticoagulation.

**Figure 5:**
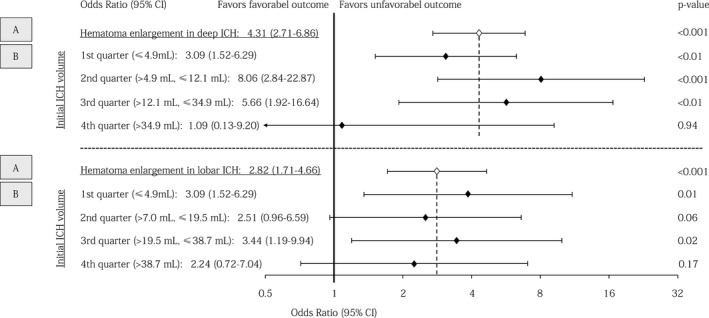
Association of hematoma enlargement with 3‐month functional outcome according to ICH location and initial hematoma volume. Forest plots showing association of hematoma enlargement with functional outcome in (A) deep and lobar ICH patients and (B) deep and lobar ICH patients in relation to initial hematoma volume split according to quartiles. Multivariable modeling in both analyses (A + B) included adjustment for relevant outcome predictors, that is, age, Glasgow Coma Scale, oral anticoagulation, initial ICH volume, and intraventricular hemorrhage. Abbreviations: CI, Confidence interval; ICH, Intracerebral hemorrhage.

In both patients with deep and lobar non‐OAC‐ICH as well as OAC‐ICH, occurrence of HE showed significant association with mortality and unfavorable outcome after 3 months, see Figure 4 for mRS distribution. Performing multivariable regression analyses in combined cohorts of non‐OAC‐ICH and OAC‐ICH‐patients, both adjusted OR for mortality and unfavorable functional outcome in case of HE were higher in patients with deep (OR, 4.62 [3.07–6.95], *P* < 0.001 and 4.31 [2.71–6.86], *P* < 0.001) compared to lobar ICH (OR, 2.41 [1.45–4.00], *P* < 0.001 and 2.82 [1.71–4.66], *P* < 0.001), Figure 5A.

To correlate the clinical relevance of HE with initial ICH size, we grouped patients according to quartiles of initial ICH volume, Figure 5B. We observed increased odds for unfavorable outcome after HE in both small‐ to medium‐sized deep and lobar ICH volumes (1st quarter, OR, deep: 3.09 [1.52–6.29], *P* < 0.01; lobar: 3.86 [1.35–11.04], *P* = 0.01). HE after initial large ICH did only significantly increased odds for mortality (4th quarter, OR, deep: 7.76 [1.91–31.58], *P* < 0.01; lobar: 2.83 [1.16–6.88], *P* = 0.02; Table S7) but not for unfavorable functional outcome in both deep and lobar ICH patients (4th quarter, OR, deep:1.09 [0.13–9.20], *P* = 0.94; lobar: 2.24 [0.72–7.04], *P* = 0.17), Figure 5B.

## Discussion

Previous studies analyzing HE with respect to ICH location were small‐sized and with limited statistical power and possibilities for adjusting imbalances given inherently heterogeneous cohorts.^12^, ^16^ To our knowledge, the present study represents the largest individual patient data analysis to date investigating HE characteristics in relation to supratentorial ICH location. While we observed no differences regarding the occurrence of HE in deep versus lobar ICH, we detected a greater extent of HE after deep VKA‐ and NOAC‐ICH. Overall, HE in deep compared to lobar ICH occurred earlier after symptom onset. Although HE was associated with worse clinical outcome specifically in those patients with small to medium initial bleedings in both locations, hematoma enlargement seems to be of greater clinical significance in deep versus lobar ICH.

Supratentorial ICH includes deep location, usually hypertension‐induced basal ganglia bleedings, compared to lobar sites, frequently caused by CAA.^13^ In addition to diverse etiologies also the patient´s clinical presentation may differ, affecting motor function more significantly in deep ICH, whereas cortical deficits may dominate in lobar ICH.^15^ Thus, deep bleedings result more frequently in worse functional outcome if latter is assessed using a scale with overrepresentation of motor functions, for example, mRS.^23^, ^31^ Yet, various randomized trials targeting HE did not distinguish between deep and lobar ICH location, and thus did not account for potential different dynamics or functional relevance of HE, overall undermining primary clinical outcome analyses. Considering HE as an continued‐ongoing bleeding facilitated by increased blood pressure levels, and altered coagulation, respectively,^5^, ^32^ prior studies aimed to reduce its occurrence to eventually prevent worsening odds for functional independence.^7^, ^8^, ^9^, ^10^, ^11^ Our findings considerably add to these studies and might assist opening avenues for stricter enrolment criteria of future hemostatic and blood pressure management trials.^7^, ^8^, ^9^, ^10^, ^11^, ^33^ Similar to the body of knowledge generated from the negative findings of thrombectomy in rather unselected ischemic stroke patients of 2013,^34^, ^35^, ^36^ these studies served as a basis for developing study designs that proved significance thereafter.^37^

Our data suggest that although the overall occurrence does not differ, HE in hypertension‐based loco‐typico bleedings, compared to lobar ICH, occurs earlier and thus may be targeted more effectively by rapid systolic blood pressure reduction. This aspect is supported by post‐hoc analyses of the ATACH‐II trial verifying reduced HE rates after early blood pressure reduction for deep ICH patients only.^8^, ^11^ In contrast to non‐OAC‐ICH, we observed a greater HE extent after OAC‐related deep ICH, hinting toward a multiplying interaction of altered hemostasis and arterial hypertension, as already suggested earlier.^3^ According to our data, interventions targeting HE in lobar ICH might also be promising at later time‐points as radiological HE detection rate showed an almost linear increase over first 24 h. However, the FAST and the TICH‐2 studies demonstrated reduced rates of HE achieved by tested hemostatic interventions, yet without translating to primary disability outcomes.^9^, ^10^ The present study results suggest that association of HE with functional outcome is more pronounced in deep compared to lobar ICH, likely due to increased damage of passing pyramidal tracts resulting in poor motor function in deep ICH.^15^ Although the likelihood of HE is known to be higher in larger ICH volumes,^5^ we documented that the initially high odds for an unfavorable outcome in larger baseline ICH will be less influenced by additional HE as compared to patients with smaller ICH volumes. Therefore, patients having “much to lose” because of small to medium initial ICH should be randomized to treatment interventions.^33^ Although prevention of HE represents a logical pursuit in overall ICH treatment, it might only produce a statistically significant clinical benefit in strictly selected patients.^33^, ^38^

The strengths of the present investigation include its large sample size allowing adequate statistical adjustment for established HE predictors, specifically ICH volume and time from symptom onset until initial and control imaging. Using multicenter real‐world data collected at a wide variety of clinical settings, including tertiary care University as well as smaller community hospitals strengthens generalizability of findings. However, several limitations of this study are obvious. First, the retrospective design reduced data quality.^3^ Second, ICH volume measurement was not done volumetrically harboring residual imprecision of HE scoring.^26^ Third, we focused on intraparenchymal HE and did not assess IVH progressions. Fourth, a routine clinical management was executed at each individual center, rather than protocoled prospective imaging examinations at certain time‐points to verify or exclude HE, thus limiting accuracy of reported time frames for HE occurrence. Fifth, contrary to the multicenter enrollment of patients with OAC‐ICH, data on patients with non‐OAC‐ICH were collected at one single university hospital, which decreases generalizability. Sixth, we utilized PSM to account for imbalances in observed confounders; however, residual confounding after matching procedure cannot be excluded.

## Conclusions

This study establishes that the occurrence of HE does not differ among deep and lobar ICH, irrespective of associated OAC. However, compared to lobar ICH, HE after deep ICH is of greater extent in presence of OAC, may occur earlier from symptom onset and seems to be of greater clinical relevance. Overall, the clinical significance is more apparent in small to medium compared to large‐sized bleedings. These data may be valuable for both routine clinical management as well as for designing future studies on hemostatic and blood pressure management aming at minimizing HE. However, further studies with improved design are needed to replicate these findings and to investigate the pathophysiological mechanisms accounting for these observations.

## Conflict of Interest

The authors have no conflicts of interest related to the contents of the manuscript.

## Authors’ Contributions

Jochen A. Sembill: Conception and design of the study, acquisition and analysis of data, and drafting of the manuscript and figures.

Joji B. Kuramatsu: Conception and design of the study, acquisition and analysis of data, and drafting a significant portion of the manuscript.

Stefan T. Gerner, Maximilian I. Sprügel, Sebastian S. Roeder, Dominik Madžar, Manuel Hagen, Philip Hoelter, Hannes Lücking, and Arnd Dörfler: Acquisition and analysis of data.

Stefan Schwab: Conception and design of the study and drafting a significant portion of the manuscript.

Hagen B. Huttner: Conception and design of the study, acquisition and analysis of data, and drafting of the manuscript.

## Supporting information


**Table S1.** Clinical characteristics of patients with deep versus lobar ICH.
**Table S2.** Clinical characteristics of patients with versus without hematoma enlargement.
**Table S3.** Clinical characteristics of patients with deep versus lobar ICH after propensity score matching.
**Table S4.** Sensitivity analyses of patients excluded and included into propensity score matched analyses.
**Table S5.** Interaction of excluded patients with hematoma enlargement according to ICH location.
**Table S6.** Location‐specific association of time from initial to control imaging with hematoma enlargement.
**Table S7.** Association of hematoma enlargement with mortality in relation to initial intracerebral hematoma volume.
**Figure S1.** Standardized mean differences and propensity scores before and after matching procedure.

## References

[1] Brouwers HB , Falcone GJ , McNamara KA , et al. CTA spot sign predicts hematoma expansion in patients with delayed presentation after intracerebral hemorrhage. Neurocrit Care 2012;17:421–428. 22878870 10.1007/s12028-012-9765-2 PMC3707619

[2] Flibotte JJ , Hagan N , O'Donnell J , et al. Warfarin, hematoma expansion, and outcome of intracerebral hemorrhage. Neurology 2004;28:1059–1064. 10.1212/01.wnl.0000138428.40673.83 15452298

[3] Kuramatsu JB , Gerner ST , Schellinger PD , et al. Anticoagulant reversal, blood pressure levels, and anticoagulant resumption in patients with anticoagulation‐related intracerebral hemorrhage. JAMA 2015;24:824–836. 10.1001/jama.2015.0846 25710659

[4] Dowlatshahi D , Yogendrakumar V , Aviv RI , et al. Small intracerebral hemorrhages have a low spot sign prevalence and are less likely to expand. Int J Stroke 2016;11:191–197. 26783310 10.1177/1747493015616635

[5] Al‐Shahi Salman R , Frantzias J , Lee RJ , et al. Absolute risk and predictors of the growth of acute spontaneous intracerebral haemorrhage: a systematic review and meta‐analysis of individual patient data. Lancet Neurol 2018;17:885–894. 30120039 10.1016/S1474-4422(18)30253-9 PMC6143589

[6] Brott T , Broderick J , Kothari R , et al. Early hemorrhage growth in patients with intracerebral hemorrhage. Stroke 1997;28:1–5. 8996478 10.1161/01.str.28.1.1

[7] Anderson CS , Heeley E , Huang Y , et al. Rapid blood‐pressure lowering in patients with acute intracerebral hemorrhage. N Engl J Med 2013;20:2355–2365. 10.1056/NEJMoa1214609 23713578

[8] Qureshi AI , Palesch YY , Barsan WG , et al. Intensive blood‐pressure lowering in patients with acute cerebral hemorrhage. N Engl J Med 2016;15:1033–1043. 10.1056/NEJMoa1603460 PMC5345109 27276234

[9] Sprigg N , Flaherty K , Appleton JP , et al. Tranexamic acid for hyperacute primary IntraCerebral Haemorrhage (TICH‐2): an international randomised, placebo‐controlled, phase 3 superiority trial. Lancet 2018;391:2107–2115. 29778325 10.1016/S0140-6736(18)31033-X PMC5976950

[10] Mayer SA , Brun NC , Begtrup K , et al. Efficacy and safety of recombinant activated factor VII for acute intracerebral hemorrhage. N Engl J Med 2008;15:2127–2137. 10.1056/NEJMoa0707534 18480205

[11] Leasure AC , Qureshi AI , Murthy SB , et al. Association of intensive blood pressure reduction with risk of hematoma expansion in patients with deep intracerebral hemorrhage. JAMA Neurol 2019;76:949. 10.1001/jamaneurol.2019.1141 PMC6515816 31081862

[12] Yogendrakumar V , Demchuk AM , Aviv RI , et al. Location of intracerebral haemorrhage predicts haematoma expansion. Eur Stroke J. 2017;2:257–263. 31008319 10.1177/2396987317715836 PMC6454825

[13] Flaherty ML , Woo D , Haverbusch M , et al. Racial variations in location and risk of intracerebral hemorrhage. Stroke 2005;36:934–937. 15790947 10.1161/01.STR.0000160756.72109.95

[14] Falcone GJ , Biffi A , Brouwers HB , et al. Predictors of hematoma volume in deep and lobar supratentorial intracerebral hemorrhage. JAMA Neurol 2013;70:988–994. 23733000 10.1001/jamaneurol.2013.98 PMC3808840

[15] Delcourt C , Sato S , Zhang S , et al. Intracerebral hemorrhage location and outcome among INTERACT2 participants. Neurology 2017;11:1408–1414. 10.1212/WNL.0000000000003771 PMC5386433 28235817

[16] Roh D , Sun CH , Murthy S , et al. Hematoma expansion differences in lobar and deep primary intracerebral hemorrhage. Neurocrit Care 2019;31:40–45. 30756318 10.1007/s12028-018-00668-2 PMC6609462

[17] Gerner ST , Kuramatsu JB , Sembill JA , et al. Association of prothrombin complex concentrate administration and hematoma enlargement in non‐vitamin K antagonist oral anticoagulant‐related intracerebral hemorrhage. Ann Neurol 2018;83:186–196. 29314216 10.1002/ana.25134

[18] Sprugel MI , Sembill JA , Kuramatsu JB , et al. Heparin for prophylaxis of venous thromboembolism in intracerebral haemorrhage. J Neurol Neurosurg Psychiatry 2019;90:783–791. 30992334 10.1136/jnnp-2018-319786

[19] Kuramatsu JB , Sembill JA , Gerner ST , et al. Management of therapeutic anticoagulation in patients with intracerebral haemorrhage and mechanical heart valves. Eur Heart J 2018;39:1709–1723. 29529259 10.1093/eurheartj/ehy056 PMC5950928

[20] Kuramatsu JB , Biffi A , Gerner ST , et al. Association of surgical hematoma evacuation vs conservative treatment with functional outcome in patients with cerebellar intracerebral hemorrhage. JAMA 2019;8:1392–1403. 10.1001/jama.2019.13014 PMC6784768 31593272

[21] Gerner ST , Kuramatsu JB , Sembill JA , et al. Characteristics in non‐vitamin K antagonist oral anticoagulant‐related intracerebral hemorrhage. Stroke 2019;50:1392–1402. 31092170 10.1161/STROKEAHA.118.023492

[22] Sprugel MI , Kuramatsu JB , Gerner ST , et al. Antiplatelet therapy in primary spontaneous and oral anticoagulation‐associated intracerebral hemorrhage. Stroke 2018;49:2621–2629. 30355188 10.1161/STROKEAHA.118.021614

[23] Sembill JA , Gerner ST , Volbers B , et al. Severity assessment in maximally treated ICH patients: the max‐ICH score. Neurology 2017;01:423–431. 10.1212/WNL.0000000000004174 28679602

[24] Kothari RU , Brott T , Broderick JP , et al. The ABCs of measuring intracerebral hemorrhage volumes. Stroke 1996;27:1304–5. 8711791 10.1161/01.str.27.8.1304

[25] Huttner HB , Steiner T , Hartmann M , et al. Comparison of ABC/2 estimation technique to computer‐assisted planimetric analysis in warfarin‐related intracerebral parenchymal hemorrhage. Stroke 2006;37:404–408. 16373654 10.1161/01.STR.0000198806.67472.5c

[26] Webb AJ , Ullman NL , Morgan TC , et al. Accuracy of the ABC/2 score for intracerebral hemorrhage: systematic review and analysis of MISTIE, CLEAR‐IVH, and CLEAR III. Stroke 2015;46:2470–2476. 26243227 10.1161/STROKEAHA.114.007343 PMC4550520

[27] Labovitz DL , Halim A , Boden‐Albala B , et al. The incidence of deep and lobar intracerebral hemorrhage in whites, blacks, and Hispanics. Neurology 2005;23:518–522. 10.1212/01.wnl.0000172915.71933.00 16116109

[28] Sembill JA , Sprugel MI , Gerner ST , et al. Influence of prior nicotine and alcohol use on functional outcome in patients after intracerebral hemorrhage. J Stroke Cerebrovasc Dis 2018;27:892–899. 29191740 10.1016/j.jstrokecerebrovasdis.2017.10.029

[29] Sembill JA , Wieser CY , Sprugel MI , et al. Initiating anticoagulant therapy after ICH is associated with patient characteristics and treatment recommendations. J Neurol 2018;265:2404–2414. 30128711 10.1007/s00415-018-9009-2

[30] Biffi A , Battey TW , Ayres AM , et al. Warfarin‐related intraventricular hemorrhage: imaging and outcome. Neurology 2011;15:1840–1846. 10.1212/WNL.0b013e3182377e12 PMC3233208 22049204

[31] Sreekrishnan A , Dearborn JL , Greer DM , et al. Intracerebral hemorrhage location and functional outcomes of patients: a systematic literature review and meta‐analysis. Neurocrit Care 2016;25:384–391. 27160888 10.1007/s12028-016-0276-4

[32] Hemphill JC 3rd , Greenberg SM , Anderson CS , et al. Guidelines for the management of spontaneous intracerebral hemorrhage: a guideline for healthcare professionals from the American Heart Association/American stroke association. Stroke 2015;46:2032–2060. 26022637 10.1161/STR.0000000000000069

[33] Tuhrim S . Intracerebral hemorrhage–improving outcome by reducing volume? N Engl J Med 2008;15:2174–2176. 10.1056/NEJMe0801856 18480212

[34] Broderick JP , Palesch YY , Demchuk AM , et al. Endovascular therapy after intravenous t‐PA versus t‐PA alone for stroke. N Engl J Med 2013;7:893–903. 10.1056/NEJMoa1214300 PMC3651875 23390923

[35] Kidwell CS , Jahan R , Gornbein J , et al. A trial of imaging selection and endovascular treatment for ischemic stroke. N Engl J Med 2013;7:914–923. 10.1056/NEJMoa1212793 PMC3690785 23394476

[36] Ciccone A , Valvassori L , Nichelatti M , et al. Endovascular treatment for acute ischemic stroke. N Engl J Med 2013;7:904–913. 10.1056/NEJMoa1213701 PMC3708480 23387822

[37] Goyal M , Menon BK , van Zwam WH , et al. Endovascular thrombectomy after large‐vessel ischaemic stroke: a meta‐analysis of individual patient data from five randomised trials. Lancet 2016;387:1723–1731. 26898852 10.1016/S0140-6736(16)00163-X

[38] Hemphill JC 3rd . Hematoma expansion in ICH: targeting epidemiology or biology? Neurocrit Care 2019;31:9–10. 31161420 10.1007/s12028-019-00752-1

