# Supplementary material for: Hematoma enlargement characteristics in deep versus lobar intracerebral hemorrhage
Source: Ann Clin Transl Neurol. 2020 Mar 4;7(3):363–74. doi: 10.1002/acn3.51001 (PMC7086015; doi:10.1002/acn3.51001)
Supplement: Supplementary file 1 — Table S1. Clinical characteristics of patients with deep versus lobar ICH. Table S2. Clinical characteristics of patients with versus without hematoma enlargement. Table S3. Clinical characteristics of patients with deep versus lobar ICH after propensity score matching. Table S4. Sensitivity analyses of patients excluded and included into propensity score matched analyses. Table S5. Interaction of excluded patients with hematoma enlargement according to ICH location. Table S6. Location‐specific association of time from initial to control imaging with hematoma enlargement. Table S7. Association of hematoma enlargement with mortality in relation to initial intracerebral hematoma volume. Figure S1. Standardized mean differences and propensity scores before and after matching procedure. [file ACN3-7-363-s001.pdf]

Supplementary material

Sembill et al.

Hematoma enlargement characteristics in deep vs lobar intracerebral hemorrhage

Content:

e-Table 1. Clinical characteristics of patients with deep vs lobar ICH

e-Table 2. Clinical characteristics of patients with vs without hematoma enlargement

e-Table 3. Clinical characteristics of patients with deep vs lobar ICH after propensity score matching

e-Figure 1. Standardized mean differences and propensity scores before and after matching procedure

e-Table 4. Sensitivity analyses of patients excluded and included into propensity score matched analyses

e-Table 5. Interaction of excluded patients with hematoma enlargement according to ICH location

e-Table 6. Location-specific association of time from initial to control imaging with hematoma enlargement

e-Table 7. Association of hematoma enlargement with mortality in relation to initial intracerebral hematoma volume

e-Table 1. Clinical characteristics of patients with deep vs lobar ICH

Characteristics	Deep (n=1106)	Lobar (n=848)	p-value
Age† (yrs)	74 (67-80)	76 (70-81)	<0.001
Sex* (♀)	425 (38.1%)	372 (43.9%)	0.02 [e]
Pre-mRS†[a]	0 (0-1)	0 (0-2)	0.06
Prior medical history			
Hypertension*	984 (89.0%)	720 (84.9%)	0.008 [e]
Diabetes Mellitus*	341 (30.8%)	246 (29.0%)	0.38
Prior ischemic stroke or TIA*	292 (26.4%)	192 (22.6%)	0.06
Prior ICH or major bleeding*	82 (7.4%)	102 (12.0%)	<0.001
Congestive heart failure*	186 (16.8%)	137 (16.2%)	0.70
Renal insufficiency*	241 (21.8%)	172 (20.3%)	0.42
Antiplatelet use*	179 (16.2%)	174 (20.5%)	0.01 [e]
Vitamin K Antagonist use*	681 (61.8%)	505 (59.6%)	0.37
NOAC use*	69 (6.2%)	38 (4.5%)	0.09
On admission status			
Glasgow coma scale‡[d]	13 (11-15)	14 (12-15)	<0.001
ICH Score‡[e]	1 (0-2)	1 (0-2)	0.08
Systolic blood pressure† (mmHg)	170 (150-190)	162 (145-181)	<0.001
Imaging			
Symptom onset – first imaging, min	156 (85-364)	291 (140-740)	<0.001
Symptom onset – second imaging, h	24 (13-36)	29 (17-52)	<0.001
ICH volume‡ (cm^3)	9.4 (3.9-18.3)	19.5 (7.0-38.7)	<0.001
Intraventricular hemorrhage*	540 (48.8%)	240 (28.3%)	<0.001
Reversal treatment (OAC patients, n=1293)			
Any reversal treatment*	725/750 (96.7%)	513/543 (94.5%)	0.05
Admission – reversal, min	95 (60-162)	107 (66-206)	0.007 [e]
PCC*	656/750 (87.5%)	445/543 (82.0%)	0.006 [e]
FFP*	32/750 (4.3%)	41/543 (7.6%)	0.01 [e]
Konakion*	601/750 (80.1%)	429/543 (79.0%)	0.62
Coagulation parameters (OAC patients, n=1293)			
INR on admission‡	2.50 (2.06-3.10)	2.60 (2.09-3.40)	0.01 [e]
1st INR after reversal‡[g]	1.26 (1.15-1.43)	1.30 (1.17-1.56)	<0.001
Length of stay (d) ‡	13 (8-19)	10 (7-15)	<0.001

* n (%); † median (Interquartile range, 25th-75th percentile)

Abbreviations: d, days; FFP, Fresh frozen plasma; h, hours; ICH, intracerebral hemorrhage; INR, international normalized ratio; m, minutes; NOAC, Non-vitamin K antagonist oral anticoagulants; OAC, Oral anticoagulant; PCC, prothrombin complex concentrate; TIA, transient ischemic attack; yrs, years.

[a] modified Rankin Scale, range 0-6, from no disability to death; [b] Glasgow coma scale, range 3-15, from coma to alertness. [c] ICH score, range 0-6, from low to high short-term mortality risk. [d] First value of in-hospital monitoring after reversal treatment, if appropriate. [e] Not significant after performance of Holm's sequential Bonferroni correction.

e-Table 2. Clinical characteristics of patients with vs without hematoma enlargement

Characteristics	Non-OAC-ICH (n=661)			OAC-ICH (n=1293)		
	**No hematoma enlargement (n=586)**	**Hematoma enlargement (n=75)**	**p-value**	**No hematoma enlargement (n=828)**	**Hematoma enlargement (n=465)**	**p-value**
Age† (yrs)	72 (63-79)	72 (57-79)	0.48	76 (71-81)	76 (70-81)	0.04 [e]
Sex* (♀)	254 (43.3%)	36 (48.0%)	0.44	336 (40.6%)	167 (35.9%)	0.10
Pre-mRS†[a]	1 (0-2)	1 (0-2)	0.32	0 (0-2)	0 (0-1)	0.31
<u>Prior medical history</u>						
Hypertension*	505 (86.2%)	62 (82.7%)	0.41	731 (88.3%)	406 (87.3%)	0.60
Diabetes Mellitus*	152 (25.9%)	21 (28.0%)	0.70	282 (34.1%)	132 (28.4%)	0.04 [e]
Prior ischemic stroke or TIA*	107 (18.3%)	10 (13.3%)	0.29	234 (28.3%)	133 (28.6%)	0.89
Prior ICH or major bleeding*	73 (12.5%)	4 (5.3%)	0.07	66 (8.0%)	41 (8.8%)	0.60
Congestive heart failure*	66 (11.3%)	10 (13.3%)	0.60	165 (19.9%)	82 (17.6%)	0.31
Renal insufficiency*	69 (11.8%)	11 (14.7%)	0.47	205 (24.8%)	128 (27.5%)	0.28
Antiplatelet use*	196 (33.4%)	25 (33.3%)	1.00	82 (9.9%)	50 (10.8%)	0.63
<u>On admission status</u>						
Glasgow coma scale†[b]	13 (11-15)	13 (8-15)	0.06	14 (12-15)	13 (11-15)	<0.001
ICH Score†[c]	1 (0-2)	1 (1-2)	0.03 [e]	1 (0-2)	1 (0-2)	0.03 [e]
Systolic blood pressure† (mmHg)	169 (149-189)	170 (155-197)	0.26	164 (145-183)	169 (148-191)	0.05 [e]
<u>Imaging</u>						
Symptom onset – first imaging† (min)	332 (156-833)	217 (112-502)	0.009 [e]	163 (88-393)	118 (80-256)	<0.001
Symptom onset – second imaging† (h)	31 (21-50)	25 (17-43)	0.009 [e]	23 (12-36)	19 (9-31)	0.001
ICH volume† (mL)	11.6 (4.2-27.4)	13.5 (3.8-29.1)	0.77	10.9 (4.9-24.0)	14.2 (5.3-35.6)	<0.001
Intraventricular hemorrhage*	258 (44.0%)	44 (58.7%)	0.02 [e]	317 (38.3%)	161 (34.6%)	0.19
<u>Coagulation parameters (VKA patients)</u>						
INR on admission‡<br>1st INR after reversal‡ [d]	N/A	N/A	N/A	2.56 (2.13-3.20)<br>1.26 (1.16-1.46)	2.70 (2.25-3.40)<br>1.34 (1.19-1.58)	0.004 [e]<br><0.001

* n (%); † median (Interquartile range, 25th-75th percentile)

Abbreviations: h, hours; ICH, intracerebral hemorrhage; INR, international normalized ratio; m, minutes; N/A, not applicable; OAC, Oral anticoagulant; TIA, transient ischemic attack; VKA, vitamin K antagonists; yrs, years.

[a] modified Rankin Scale, range 0-6, from no disability to death; [b] Glasgow coma scale, range 3-15, from coma to alertness. [c] ICH score, range 0-6, from low to high short-term mortality risk. [d] First value of in-hospital monitoring after reversal treatment, if appropriate. [e] Not significant after performance of Holm's sequential Bonferroni correction.

e-Table 3. Clinical characteristics of patients with deep vs lobar ICH after propensity score matching

Characteristics	Non-OAC-ICH (n=270)			OAC-ICH (n=314)		
	**Deep (n=135)**	**Lobar (n=135)**	**p-value**	**Deep (n=157)**	**Lobar (n=157)**	**p-value**
Age† (yrs)	70 (60-79)	73 (65-78)	0.10	77 (70-83)	75 (70-81)	0.27
Sex* (♀)	62 (45.9%)	72 (53.3%)	0.22	60 (38.2%)	58 (36.9%)	0.82
Pre-mRS†[a]	1 (0-2)	1 (0-2)	0.42	0 (0-1)	0 (0-1)	0.88
Prior medical history						
Hypertension*	121 (89.6%)	107 (79.3%)	0.02 [e]	142 (90.4%)	136 (86.6%)	0.29
Diabetes Mellitus*	46 (34.1%)	34 (25.2%)	0.11	54 (34.4%)	50 (31.8%)	0.63
Prior ischemic stroke or TIA*	30 (22.2%)	27 (20.0%)	0.66	56 (35.7%)	49 (31.2%)	0.40
Prior ICH or major bleeding*	14 (10.4%)	13 (9.6%)	0.84	15 (9.6%)	14 (8.9%)	0.84
Congestive heart failure*	17 (12.6%)	9 (6.7%)	0.10	19 (12.1%)	29 (18.5%)	0.12
Renal insufficiency*	19 (14.1%)	8 (5.9%)	0.03 [e]	39 (24.8%)	42 (26.8%)	0.70
Antiplatelet use*	39 (28.9%)	50 (37.0%)	0.15	22 (14.0%)	12 (7.6%)	0.07
Vitamin K Antagonist use*	N/A	N/A	N/A	142 (90.4%)	141 (89.8%)	0.84
NOAC use*	N/A	N/A	N/A	15 (9.6%)	16 (10.2%)	0.84
On admission status						
Glasgow coma scale†[b]	13 (11-15)	14 (11-15)	0.56	14 (12-15)	14 (12-15)	0.83
ICH Score†[c]	1 (1-2)	1 (0-2)	<0.001	1 (0-2)	1 (0-2)	0.35
Systolic blood pressure† (mmHg)	169 (148-190)	170 (150-186)	0.91	170 (140-187)	164 (150-187)	0.97
Imaging						
Symptom onset – first imaging† (min)	280 (11-538)	289 (155-502)	0.94	139 (85-282)	143 (87-257)	0.86
Symptom onset – second imaging† (h)	30 (22-43)	29 (19-41)	0.39	23 (10-36)	23 (11-33)	0.91
ICH volume† (mL)	10.7 (3.4-24.3)	12.8 (4.2-25.2)	0.60	11.7 (4.6-22.4)	10.9 (4.6-24.4)	0.96
Intraventricular hemorrhage*	86 (63.7%)	33 (24.4%)	<0.001	60 (38.2%)	27 (17.2%)	<0.001
Reversal treatment						
Any reversal treatment*	N/A	N/A	N/A	151 (96.2%)	148 (94.3%)	0.43
Admission – reversal† (min)	N/A	N/A	N/A	95 (52-146)	95 (60-169)	0.45
PCC*	N/A	N/A	N/A	138 (87.9%)	135 (86.0%)	0.62
FFP*	N/A	N/A	N/A	11 (7.0%)	11 (7.0%)	1.00
Konakion*	N/A	N/A	N/A	122 (77.7%)	125 (79.6%)	0.68
Coagulation parameters						

INR on admission‡	1.01 (0.97-1.09)	1.03 (0.97-1.07)	0.74	2.53 (2.10-3.19)	2.50 (2.01-3.32)	0.96
1st INR after reversal‡ [d]	N/A	N/A	N/A	1.28 (1.17-1.42)	1.30 (1.16-1.50)	0.44
Length of stay (d) ‡	14 (8-20)	9 (7-14)	<0.001	11 (6-17)	10 (7-16)	0.88

* n (%); † median (Interquartile range, 25th-75th percentile)

Abbreviations: FFP, Fresh frozen plasma; h, hours; ICH, intracerebral hemorrhage; INR, international normalized ratio; m, minutes; N/A, not applicable; NOAC, Non-vitamin K antagonist oral anticoagulants; OAC, Oral anticoagulant; PCC, prothrombin complex concentrate; TIA, transient ischemic attack; yrs, years.

[a] modified Rankin Scale, range 0-6, from no disability to death; [b] Glasgow coma scale, range 3-15, from coma to alertness. [c] ICH score, range 0-6, from low to high short-term mortality risk. [d] First value of in-hospital monitoring after reversal treatment, if appropriate. [e] Not significant after performance of Holm's sequential Bonferroni correction.

e-Figure 1. Standardized mean differences and propensity scores before and after matching procedure

Non-OAC-ICH | **OAC-ICH**

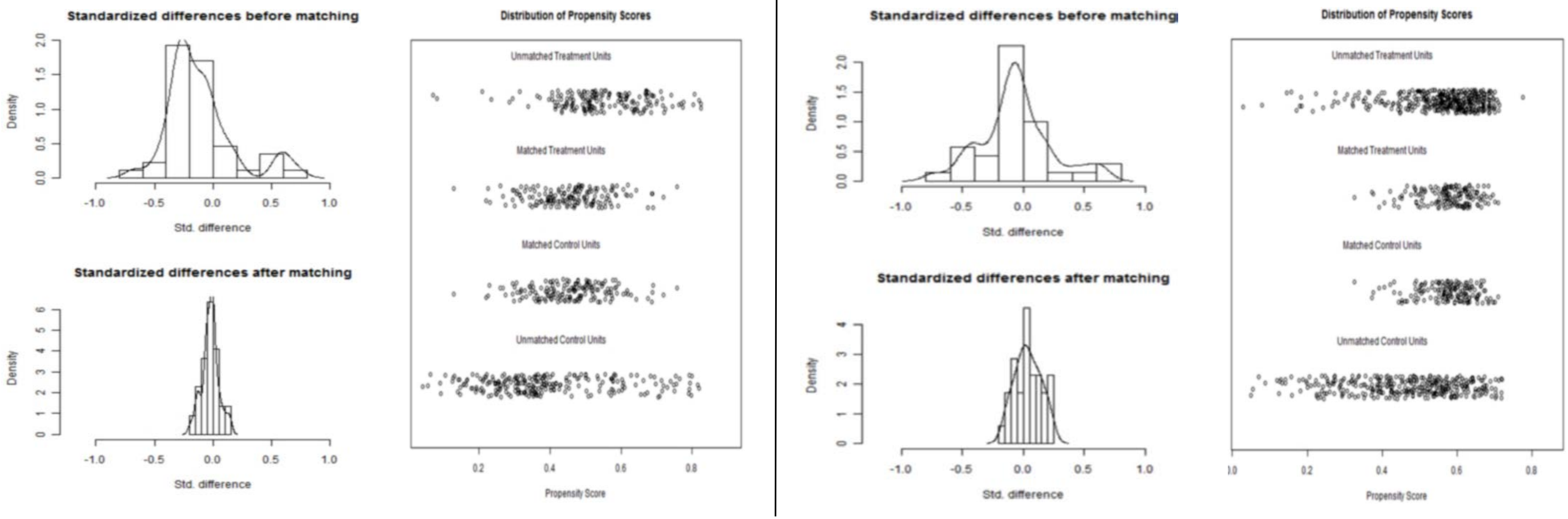

Standardized mean differences of parameters used for calculating of propensity scores as well as distribution of propensity scores before and after the matching procedure.
Abbreviations: ICH, intracerebral hemorrhage; OAC, Oral anticoagulant.

eTable 4. Sensitivity analyses of patients excluded and included into propensity-matched analyses

	Comparison of deep ICH patients			Comparison of lobar ICH patients		
**Characteristics**	**Excluded (n=814)**	**Included (n=292)**	**p-value**	**Excluded (n=556)**	**Included (n=292)**	**p-value**
Age† (yrs)	74 (67-80)	73 (66-80)	0.38	76 (70-81)	76 (68-81)	0.31
Sex* (♀)	299 (36.7%)	122 (41.8%)	0.13	242 (43.5%)	130 (44.5%)	0.780
Pre-mRS†[a]	0 (0-1)	0 (0-1)	0.65	0 (0-2)	0 (0-2)	0.58
Prior medical history						
Hypertension*	721 (88.6%)	263 (90.1%)	0.48	477 (85.8%)	243 (83.2%)	0.32
Diabetes Mellitus*	241 (29.6%)	100 (34.2%)	0.14	162 (29.1%)	84 (28.8%)	0.92
Prior ischemic stroke or TIA*	206 (25.3%)	86 (29.5%)	0.17	116 (20.9%)	76 (26.0%)	0.09
Prior ICH or major bleeding*	53 (6.5%)	29 (9.9%)	0.06	75 (13.5%)	27 (9.2%)	0.07
Congestive heart failure*	150 (18.4%)	36 (12.3%)	0.02 [e]	99 (17.8%)	38 (13.0%)	0.07
Renal insufficiency*	183 (22.5%)	58 (19.9%)	0.35	122 (21.9%)	50 (17.1%)	0.10
Antiplatelet use*	118 (14.5%)	61 (20.9%)	0.01 [e]	112 (20.1%)	62 (21.2%)	0.71
Vitamin K Antagonist use*	539 (66.2%)	142 (48.6%)	<0.001	364 (65.5%)	141 (48.3%)	<0.001
NOAC use*	54 (6.6%)	15 (5.1%)	0.37	22 (4.0%)	16 (5.5%)	0.31
On admission status						
Glasgow coma scale†[b]	13 (10-15)	14 (11-15)	0.007 [e]	14 (12-15)	14 (12-15)	0.44
ICH Score†[c]	1 (0-2)	1 (0-2)	0.76	1 (0-2)	1 (0-2)	<0.001
Systolic blood pressure† (mmHg)	170 (150-192)	170 (146-190)	0.26	160 (142-180)	169 (150-186)	0.001
Imaging						
Symptom onset – first imaging† (min)	140 (81-342)	189 (93-395)	0.007 [e]	445 (177-1365)	204 (106-359)	<0.001
Symptom onset – second imaging† (h)	22 (12-35)	26 (17-38)	0.002 [e]	34 (20-64)	26 (15-39)	<0.001
ICH volume† (mL)	8.9 (3.9-17.1)	11.4 (4.2-23.1)	0.006 [e]	24.0 (10.2-46.2)	11.3 (4.4-24.4)	<0.001
Intraventricular hemorrhage*	394 (48.4%)	146 (50.0%)	0.64	180 (32.4%)	60 (20.5%)	<0.001
Reversal treatment [f]						
Any reversal treatment*	574/593 (96.8%)	151/157 (96.2%)	0.70	365/386 (94.6%)	148/157 (94.3%)	0.890
Admission – reversal† (min)	95 (60-170)	95 (52-146)	0.37	116 (68-217)	95 (60-169)	0.04 [e]
PCC*	518/593 (87.4%)	138/157 (87.9%)	0.86	310/386 (80.3%)	135/157 (86.0%)	0.12
FFP*	21/593 (3.5%)	11/157 (7.0%)	0.06	30/386 (7.8%)	11/157 (7.0%)	0.76
Konakion*	479/539 (88.9%)	122/142 (85.9%)	0.33	304/364 (83.5%)	125/141 (88.7%)	0.15

Coagulation parameters [f]						
INR on admission‡	2.50 (2.05-3.06)	2.53 (2.10-3.19)	0.49	2.66 (2.10-3.44)	2.50 (2.01-3.32)	0.25
1st INR after reversal‡ [d]	1.25 (1.14-1.43)	1.28 (1.17-1.42)	0.40	1.30 (1.18-1.57)	1.30 (1.16-1.50)	0.39
Length of stay (d) ‡	13 (8-19)	12 (7-19)	0.22	11 (7-16)	10 (7-14)	0.40

* n (%); † median (Interquartile range, 25th-75th percentile)

Abbreviations: BP, blood pressure; d, days; FFP, Fresh frozen plasma; h, hours; ICH, intracerebral hemorrhage; INR, international normalized ratio; m, minutes; N/A, not applicable; PCC, prothrombin complex concentrate; TIA, transient ischemic attack; yrs, years.

[a] modified Rankin Scale, range 0-6, from no disability to death; [b] Glasgow coma scale, range 3-15, from coma to alertness. [c] ICH score, range 0-6, from low to high short-term mortality risk. [d] First value of in-hospital monitoring after reversal treatment, if appropriate. [e] Not significant after performance of Holm's sequential Bonferroni correction. [f] Reversal treatment and coagulation parameters are shown for patients using oral anticoagulation only.

eTable 5. Interaction of excluded patients with hematoma enlargement according to ICH location

Hematoma enlargement	Non-adjusted			Adjusted	
	Present	Absent	p-value	Adjusted odds ratio (95% CI)	p-value
Deep ICH patients					
Excluded	237 (29.1%)	577 (70.9%)	0.15	0.804 (0.568-1.138)	0.22
Included	72 (24.7%)	220 (75.3%)			
Lobar ICH patients					
Excluded	153 (27.5%)	403 (72.5%)	0.81	1.111 (0.711-1.736)	0.64
Included	78 (26.7%)	214 (73.3%)			
Interaction of deep*excluded-patients on hematoma enlargement				0.831 (0.608-1.137)	0.25

Analyses of occurrence of hematoma enlargement (primary outcome) regarding the interaction of ICH location with inclusion of patients into propensity-matched analyses. Displayed are unadjusted frequency distributions, adjusted odds ratios, and p-values for the interaction of ICH location with excluded patients (deep*excluded). Multivariate models were adjusted for ICH volume, time from symptom onset until first and until second imaging, age, Glasgow Coma Scale, and prior anticoagulation status to calculate adjusted odds ratios.

e-Table 6. Location-specific association of time from initial to control imaging with hematoma enlargement

**Multivariate - parameters**	**Adjusted OR (95% CI) – Hematoma enlargement**	***p*-value**
Deep ICH		
Time 1st – 2nd imaging [hours]	0.999 (0.995-1.002)	0.46
Lobar ICH		
Time 1st – 2nd imaging [hours]	0.998 (0.993-1.003)	0.37

Multivariable modeling was adjusted for relevant baseline characteristics, i.e. age, oral anticoagulants, antiplatelets, Glasgow com scale, ICH volume and intraventricular hemorrhage.

Abbreviations: CI, Confidence interval; ICH, Intracerebral hematoma; OR, Odds Ratio.

e-Table 7. Association of hematoma enlargement with mortality in relation to initial intracerebral hematoma volume

**Hematoma enlargement in deep ICH according to quartiles of ICH volume**	**Adjusted OR (95% CI) – Mortality**	**p-value**
Overall	4.62 (3.07-6.95)	<0.001
1st quarter (≤4.9 mL)	9.15 (3.61-23.19)	<0.001
2nd quarter (>4.9 mL, ≤12.1 mL)	3.42 (1.56-7.51)	<0.01
3rd quarter (>12.1 mL, ≤34.9 mL)	3.63 (1.76-7.49)	<0.001
4th quarter (>34.9 mL)	7.76 (1.91-31.58)	<0.01
**Hematoma enlargement in lobar ICH according to quartiles of ICH volume**		
Overall	2.41 (1.45-4.00)	<0.001
1st quarter (≤7.0 mL)	4.32 (0.87-21.57)	0.07
2nd quarter (>7.0 mL, ≤19.5 mL)	1.99 (0.61-6.52)	0.25
3rd quarter (>19.5 mL, ≤38.7 mL)	1.88 (0.73-4.83)	0.19
4th quarter (>38.7 mL)	2.83 (1.16-6.88)	0.02

Influence of hematoma enlargement (defined as hematoma volume increase of 33% or 6mL) on functional mortality with respect to initial ICH volume split according to quartiles. Adjusted OR were calculated by multivariable modeling adjusted for relevant outcome predictors, i.e. age, Glasgow Coma Scale, oral anticoagulation, initial ICH volume, and intraventricular hemorrhage.

Abbreviations: CI, Confidence interval; ICH, Intracerebral hematoma; OR, Odds Ratio.
